# Usability of *Learning Moment*: Features of an E-learning Tool That Maximize Adoption by Students

**DOI:** 10.5811/westjem.2019.6.42657

**Published:** 2019-12-09

**Authors:** Andrew Chu, Dea Biancarelli, Mari-Lynn Drainoni, James H. Liu, Jeffrey I. Schneider, Ryan Sullivan, Alexander Y. Sheng

**Affiliations:** * Boston University School of Medicine, Boston, Massachusetts; † Boston University School of Public Health, Department of Health Law, Policy and Management, Boston, Massachusetts; ‡ Boston University School of Medicine, Evans Center for Implementation and Improvement Sciences, Boston, Massachusetts; § Boston University School of Medicine, Section of Infectious Diseases, Department of Medicine, Boston, Massachusetts; ¶ Edith Nourse Rogers Memorial Veterans Hospital, Center for Healthcare Organization and Implementation Research, Bedford, Massachusetts; || Boston Medical Center, Department of Emergency Medicine, Boston, Massachusetts; # Lawrence General Hospital, Emergency Center, Lawrence, Massachusetts

## Abstract

**Introduction:**

E-learning is widely used in medical education. To maximize the potential of E-learning tools, every effort should be made to encourage adoption by optimizing usability. We created Learning Moment (LM), a web-based application that integrates principles of asynchronous learning and learning portfolios into a platform on which students can document and share learning experiences that occur during clinical work. We sought to evaluate the usability of LM and identify features that optimize adoption by users.

**Methods:**

We implemented LM in August 2016 at a busy, urban, tertiary care emergency department that hosts an emergency medicine residency, robust third and fourth year medical student clerkships as well as a physician assistant student rotation. We conducted a single-center, mix-methods study using the System Usability Scale (SUS) questionnaire and qualitative interviews. We sent e-mail invitations with subsequent reminders to all students who rotated in our emergency medicine clerkship from August 2016 to April 2017 to complete the SUS questionnaire anonymously and to participate in qualitative interviews. We employed purposive sampling to recruit students who used LM during their rotation to participate in our qualitative interviews. We conducted semi-structured interviews with 13 participants (10 individual interviews and one 3-person group interview) between January and March 2017 using an ethnographic approach and utilized a general inductive method to analyze and code for potential themes.

**Results:**

Thirty of the seventy students invited to participate completed the SUS questionnaire (Response rate of 42.8%). The mean SUS score is 80.9 (SD 18.2, 80% CI 76.5 – 85.3). The internal consistency of the responses achieved the Cronbach’s Alpha of 0.95. The participants stressed the importance of the following in the adoption of LM: maximal simplicity and usability, compatibility with learning preferences, and department-wide acceptance and integration.

**Conclusion:**

The overall perceived usability of LM was high. Our qualitative data revealed important implications for future designers to maximize adoption: include target users in every step of the design and development process to maximize simplicity and usability; build features that cater to a diversity of learning preferences; involve the entire department and find ways to incorporate the tool into the educational infrastructure and daily workflow.

## INTRODUCTION

E-learning describes systems that are capable of storing, managing, or modifying educational content, while also facilitating interaction between participants as they assimilate and input data.^1^ E-learning is widely used in medical education, across various specialties, educational settings, and training levels.^2^

To maximize the potential of E-learning tools, effective user-interface design is crucial to making an educational impact on the target learner population. Every effort should be made to optimize usability and reduce complexity to encourage adoption.^3^ The benefits of E-learning occur when features are effectively applied, deemed useful, and compatible with learning processes of users.^4^

While the definition of usability varies according to field of research, it is generally understood as “the capacity a system has to offer to the user in carrying out of tasks, in an effective, efficient, and satisfactory manner.”^1^ Usability of E-learning tools has been explored in various scientific disciplines from ergonomics, computer science, to design and education.^1^ In these studies, usability is often evaluated in terms of knowledge, attitudes, skills, and online activity, each of which provides an incomplete depiction of overall usability.^5^–^7^ There is paucity of literature evaluating usability of E-learning platforms using more comprehensive, validated assessment tools within medical education; and even fewer studies identifying the features that promote adoption of these E-learning tools.

We created *Learning Moment* (LM),^8^,^9^ a web-based application that integrates principles of asynchronous learning^10^,^11^ and learning portfolios^12^ to provide a platform on which students can document and share learning experiences that occur during clinical work. As described in our previous research, our intention was to optimize the experiential learning process for our students in the emergency department (ED).^8^,^9^ Understanding the importance of a learner-centered model of instructional design, our goals for this study were to evaluate the usability of LM and identify features that enhance adoption by users.

## METHODS

### Design and Implementation

In depth description of educational goals, theoretical foundation, design, implementation, utilization, sustainability, and learner experiences of LM are detailed elsewhere.^8^,^9^ In brief, Kolb’s 4-part experiential learning model (concrete experience, reflective observation, abstract conceptualization, and active experimentation) is one of the foremost experiential learning theories.^13^ Most clinical learning environments, like our ED, offers learning experiences and chances to experiment. However, they rarely provide structured opportunities for reflection and abstract conceptualization. LM fulfills these gaps to help students learn better in the clinical setting.^8^,^9^

**Educational Research Capsule Summary**

What do we already know about this issue?

*While E-learning is widely used in medical education, few studies exist to evaluate the usability of E-learning tools or identify the features that promote their adoption*.

What was the research question?

*We sought to evaluate the usability of* Learning Moment *and identify key features that optimize adoption by users*.

What was the major finding of the study?

*The usability of* Learning Moment *was high. Participants underscored three important themes that encouraged use and adoption*.

How does this improve population health?

Learning moment *features that promote usability and adoption, along with our design and implementation experiences, may be useful for other E-learning designers in medical education*.

LM (https://www.learningmoment.org/) allows students to conveniently record “learning moments” (defined as student self-identified learning experiences), highlighting the take-away “learning pearls.” The goal of LM was to provide students with a physical and mental space to synthesize experiences into coherent thoughts, thus enhancing understanding and retention through self-reflection and abstract conceptualization.^14^ By encouraging the sharing of “learning moments,” LM generates a searchable and shareable repository of useful, practical, high yield educational content^8^ that can be used for vicarious learning in the form of a “Community Feed.”^15^ Our intention was to build and support a community of practice, both live and virtual, to facilitate knowledge sharing.^16^,^17^ A three-member faculty panel reviewed the “learning moments” to ensure content validity and Health Insurance Portability and Accountability Act (HIPAA) compliance. Experienced clinical faculty led monthly in-person “*Learning Moment* Reflection” small groups with students to further discuss and expand upon the ”learning moments” logged during their rotation. Through this process, students have further opportunities to incorporate key components of Kolb’s experiential learning cycle^13^ (reflection and abstract conceptualization in particular) that are frequently absent in the bustle of today’s clinical learning environment.

We implemented LM in August 2016 at a busy (annual volume in excess of 130,000 visits), urban, tertiary care ED that hosts an emergency medicine (EM) residency, robust third and fourth year medical student clerkships as well as a physician assistant (PA) student rotation. Students were introduced to LM during their initial rotation orientation session. Participation in LM was entirely voluntary and did not affect their grade or evaluations in any way.

Within the first six months after implementation, 42 out of 53 (79.2%) students who rotated in our EM clerkship logged at least one “learning moment” for a total of 323 “learning moments” logged. These results, along with the distribution of number of “learning moments” logged by students are described elsewhere.^9^ Students have logged more than 1000 “learning moments” after 16 months of implementation, indicating continued sustainability.^8^

### Study Design and Recruitment

We conducted a single-center, mix-methods study using the System Usability Scale (SUS) questionnaire and qualitative interviews. Described as the “quick and dirty” scale that is both short and reliable, the SUS is the most widely used questionnaire for measurement of perceived usability of digital tools, including software and websites.^18^,^19^ Having been referenced in over 1,300 articles and publications, the SUS is currently the industry standard because it is easy to administer, produces reliable results even with small sample sizes, and is a validated tool for differentiating usable and unusable systems.^18^,^19^

We sent e-mail invitations with subsequent reminders to all third and fourth year medical students and PA students who rotated in our EM clerkship from August 2016 to April 2017 to complete the SUS questionnaire anonymously and to participate in qualitative interviews, regardless of the extent to which they utilized the LM platform. In addition to email invitations, we employed purposive sampling to recruit medical students who used LM during their rotation to participate in our qualitative user interviews. Our Institutional Review Board deemed our study to be exempt.

### Data Collection Procedures

For the SUS, we distributed the questionnaire and collected data using REDCap, an electronic data capture tool. We conducted semi-structured interviews with 13 participants, including 10 individual interviews and one three-person group interview, between January and March 2017. We conducted seven interviews in person, and six by telephone due to difficulty arranging face-to-face meetings. In person interviews were conducted in medical school classrooms and departmental conference rooms. We conducted interviews until we reached thematic saturation^20^ as the last several interviews yielded no additional patterns or themes. A single researcher and coauthor (AC) conducted and audio-taped all interviews using the same interview guide (Supplemental File). Individual interviews lasted between 5 and 20 minutes with a mean and median of 15 minutes and 16 minutes respectively. The three-person group interview was 26 minutes in duration.

### Data Analysis

SUS questionnaire results were compiled in aggregate and descriptive statistics were presented as frequencies. Cronbach’s alpha was used to measure the internal consistency of the questionnaire items. All questionnaire data analyses were performed using SAS v9.4 (Cary, NC). For the items of the SUS, the score was calculated using Brooke’s standard scoring method.^19^

After each qualitative interview was completed, the researcher and coauthor (AC) who conducted the interviews transcribed the audio recording verbatim. We reviewed all transcribed interviews to ensure accuracy. For analysis, we employed standard qualitative research methods using the principles of grounded theory.^21^,^22^ We coded the data inductively to generate a unified, theoretical explanation of features that would optimize adoption by users. Two coauthors (Andrew Chu and Dea Biancarelli) trained in qualitative research methods coded and generated common themes through consensus and discussion. The two co-authors initially individually reviewed a subset of transcripts and met to create an initial codebook of emerging themes. Chu and Biancarelli then applied the initial codebook to another subset of transcripts, refining and finalizing the codebook for a ‘better fit’ for the data. They applied the finalized version of the codebook to all the transcripts using qualitative software package Nvivo (QRS International, Doncaster, Victoria, Australia). After transcripts were coded, they further convened to analyze data and determine key themes users described in regard to usability and features that optimize adoption.

## RESULTS

### System Usability Scale

Thirty of the seventy students invited to participate after having rotated in our EM clerkship during the study period completed the SUS questionnaire (Response rate of 42.8%). The detailed participant demographics are listed in Table 1.

**Figure 1:**
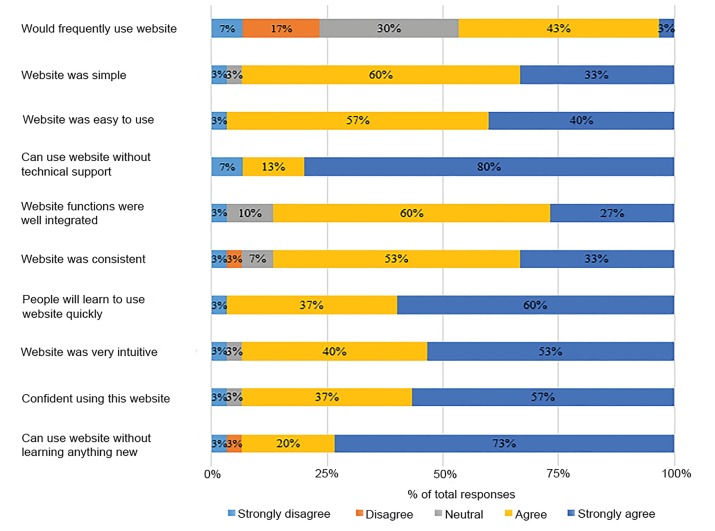
System Usability Scale questionnaire responses. Cronbach’s Alpha=0.95

The mean SUS score is 80.9 (SD 18.2, 80% confidence interval [CI], 76.5 – 85.3). The internal consistency of the responses achieved a Cronbach’s Alpha of 0.95. While the vast majority of participants answered positively (“strongly agree” or “agree”) to the questions on the SUS, only 46% reported that they “would frequently use the website” (Figure 1).

### Qualitative Interviews

Thirteen medical students (five in their third year (MS-3) and eight in their fourth year (MS-4)) voluntarily participated in our qualitative interviews. Five of the 13 (38%) students intended to pursue EM as their chosen field of specialty. No PA students volunteered to participate. Detailed demographics of participants are shown in Table 2.

In regard to features that increased the adoption of LM by users, our participants stressed the importance of the following: maximal simplicity and usability, compatibility with learning preferences, and department-wide acceptance and integration.

#### Theme 1: Maximal Simplicity and Usability

LM’s simplicity of design and high usability was lauded by LM users. Student described LM as an easy-to-use and intuitive way to reinforce learning points.

*“I like the sort of ****minimalist style**** you guys used. I love that. You know, it makes ****it pleasant and makes it useful and easy****.”- Student 6*

Any steps perceived as extraneous felt overly burdensome and disengaged students. Attitudes were shaped by time scarcity, alternative learning tools, and competing priorities.

*“****But [optional entry fields unrelated to the learning pearl such as location of learning, time of day, etc.] puts a burden on the user…to input all these other fields.”**** – Student 5*

*“But I felt that ****it was a little cumbersome just to report [optional entry fields]at times through the website****… I just felt like there were too many questions… ****Does it really matter?****” – Student 8*

#### Theme 2: Compatibility With Learning Preferences

Students explained that their learning preference greatly influenced whether or not they would utilize LM as an E-Learning tool. Many students embraced the brevity of clinical pearls. The concise and high-yield format of pearls was described as useful and easy to engage with by most students.

*“I feel like ****putting your thought into a concise kind of straightforward, like, bullet point helps you remember it****.” – Student 10*

*“Sort of building off of that, ****I also noticed the character limit, and it reminded me ‘Oh, keep this short and sweet’ and I think that helps for other people who want to go through other users’ learning moments. To go through it and be like, ‘Oh, that’s a nice little factoid, that’s a nice little tidbit.’**** And then there’s an area where like, ‘Oh, what did the patient present with, and what was the case?’ If you wanted to go through that and get more of a background, you have that ability to do that. ****So it was a nice way of presenting information in a short, sweet way, and then having an area for a little more thought and background****.” – Student 9*

However, others felt this approach was incompatible with their learning preference – that pearls were too short, too disconnected, and/or unrelated in subject matter.

***“****But a lot of people posting abbreviated learning moments****. It’s hard to learn something so significant from a one sentence thing, at least through the way I learn****. I just found better ways to learn, and Learning Moment is not one of them.” – Student 5*

*“The problem that would pose for me is that ****learning through Learning Moment is very fragmented****, right? Like, one pearl will be about the care of an alcoholic, and the next one will be about sepsis. ****I want to learn about one subject at one time and then move onto the next****.” – Student 5*

#### Theme 3: Department-wide Acceptance and Integration

Students perceived greater utility of LM the more it was used by their peers. Without peer engagement in LM, students became less interested in utilizing it as a learning tool. Students were more willing to use LM if valued by the entire department, especially when faculty and residents would integrate LM into daily workflow and didactics.

*“****You need a lot of buy-in for it to be good****… if I were using that on every single rotation, or if it were in my residency and everyone in my residency was using it…I would totally use it, because I think it’s a good tool. ****If everybody’s using it or is using it consistently throughout the year, I would totally use it.”**** – Student 7*

*“I think ****the purpose of the learning moment was to encourage an environment of teaching****. So not only was it to have students and residents reflect on things that they learned during their shift. Maybe it was also to ****encourage attendings and more senior providers to teach more**** and provide those learning moments for students on shift.”- Student 12*

*“I think ****if it was part of the curriculum where I was, it would be useful. I don’t think if I was just doing it my own thing that I would use it****.”- Student 2*

## DISCUSSION

E-learning as an educational adjunct has gained widespread popularity in various health profession education settings.^3^, ^23^, ^24^ When creating online educational programs, developers must adhere to sound educational principles that foster effective learning.^25^ We designed LM on the basis of Kolb’s experiential learning cycle,^13^ asynchronous learning,^10^ and learning portfolios^12^ essentially as an E-portfolio.^9^ Such web-based learning portfolios have been shown to enhance student motivation by students and teachers.^26^ The online format provides additional transparency and ease of administration.^27^ LM is unique among E-learning platforms in that it was created to optimize experiential learning specifically in a clinical environment.^8^,^9^

In addition to achieving a high degree of internal consistency of the responses with a Cronbach’s Alpha of 0.95, LM’s mean SUS score of 80.9 (SD 18.2, 80% CI, 76.5 – 85.3) lies in the 90th percentile when compared to other digital products. In other words, LM achieved a much higher level of perceived usability when compared to benchmarks derived from thousands of individual SUS scores and hundreds of systems, for which the average SUS score is 68, SD 12.5.^19^ Considering that a “good” SUS score is anything about a 76, the LM mean SUS score of 80.9, which received an “A” grade according to Sauro and Lewis,^19^ would receive the adjective of “excellent” per Bangor et al.^28^ While we acknowledge that such comparison has its limitations considering the heterogeneity within available E-learning products out there in terms of product goal, design, and audience, the SUS is nevertheless the industry standard specifically developed and validated for the purpose of comparing usability among digital products.^18^–^19^

Despite a robust overall SUS score, only 46% of our learners “would frequently use the website” according to the first question of the SUS (Figure 1). We believe that this may be due to the lack of significant downtime during the shift in the bustling environment of our ED for learners to document “learning moments” as well as incomplete buy-in to support LM by the department as a whole. After all, usability is necessary but not sufficient to ensure usage. Nevertheless, our actual usage data from our previous work demonstrate that LM is being used frequently.^8^,^9^

Our insights gleaned from the qualitative data can be invaluable for future designers who seek to maximize adoption. While the qualitative feedback for LM was overwhelmingly positive, few negative opinions that were expressed also provides invaluable lessons for us as E-learning designers.

In our qualitative user interviews, participants reiterated the importance of maximal simplicity and usability. Early in our conceptual design phase, we invited medical student and residents to brainstorm ideas that they believed would make the LM interface more user-friendly. Our efforts were rewarded with consistently positive usability results from both the SUS data and qualitative interviews.

Students favored the concise and high-yield nature of the learning pearls made available on LM. However, complaints from students regarding LM were related to the overly brief and random nature of learning pearls that were being logged and shared on our platform. In essence, LM did not accommodate their specific learning preferences. Despite the lack of evidence to support the existence of “learning styles” (e.g., visual, auditory, converger),^29^ learners nevertheless have their own preferred methods of learning. And matching of pedagogy to learner preferences is still recommended.^30^ In our quest to maximize simplicity and usability, we failed to anticipate the desire for some students to learn in a more comprehensive and systematic manner. Integrating the needs of various learning preferences is likely a worthwhile endeavor for future designers of E-learning.^31^ For instance, additional features that sort “learning moments” into specific diseases or organ systems would better accommodate those who prefer to learn in a more systematic fashion.

Additionally, our participants noted the significant roles that department-wide acceptance and integration significantly affected adoption of LM as an E-learning tool. Similar to previously study, community engagement and interaction matters.^32^ While E-learning can potentially reduce the need for in person didactics, it cannot replace face-to-face interaction, as students consider traditional teaching to be the foundation of their education.^6^

## LIMITATIONS

Our study has several important limitations. First, our results are limited by a response rate of 42.8%. Similarly, the sample size for qualitative interviews was small with only 13 voluntary interview participants. Nevertheless, our qualitative interviews reached thematic saturation.^20^ Students who self-selected to participate in the study may have strong positive or negative views towards LM, thus subjecting our results to participation bias. Although our recruiting e-mail describing the voluntary nature of participation, in which we stressed that participation would not affect their grade or ranking for residency application in any way, participants may have been motivated to report positive experiences with LM, thus biasing our results. The generalizability of our experience may be limited by the fact that not all E-learning tools are the same. Nevertheless, important lessons can be gleamed from LM, especially when our study is one of the first to use an industry-standard, validated tool such as the SUS in evaluation of an E-learning tool in medical education. Lastly, supplementing quantitative findings with qualitative data in a mixed methods approach as we have done in our study has been used previously and described as the best option to evaluate usability of E-learning.^1^,^33^

## Supplementary Information



## Figures and Tables

**Table 1:** System Usability Scale questionnaire participants.

Characteristics	n (%)
Discipline	
Medical student	28 (93)
Physician assistant student	2 (7)
Level of Training (medical students)	
MS-3	13 (46)
MS-4	15 (54)
Intended Future Specialty*	
Emergency medicine	16 (55)
Other	13 (45)

*MS*, medical student year.

**Table 2:** Qualitative interviewee characteristics (N=13).

Characteristics	n (%)
Year	
MS-3	5 (38.5)
MS-4	8 (61.5)
Gender	
Female	11 (84.6)
Male	2 (15.4)
Intended future specialty	
Emergency medicine	5 (38.5)
Other/unsure	8 (61.5)

*MS*, medical student year.
